# Effect of dietary fat type on intestinal digestibility of fatty acids, fatty acid profiles of breast meat and abdominal fat, and mRNA expression of lipid-related genes in broiler chickens

**DOI:** 10.1371/journal.pone.0196035

**Published:** 2018-04-19

**Authors:** Miloš Skřivan, Milan Marounek, Michaela Englmaierová, Ladislav Čermák, Jana Vlčková, Eva Skřivanová

**Affiliations:** 1 Department of Nutrition Physiology and Animal Product Quality, Institute of Animal Science, Prague-Uhrineves, Czech Republic; 2 Department of Microbiology, Nutrition and Dietetics, Faculty of Agrobiology, Food and Natural Resources, Czech University of Life Sciences Prague, Prague, Czech Republic; University of Illinois, UNITED STATES

## Abstract

A group of 240-day-old Ross cockerels were used in a 4-week experiment to assess the effect of the fat type on the intestinal digestibility of fatty acids (FAs), the FA profiles of breast meat and abdominal fat, and the mRNA expression of six hepatic lipid-related genes. Experimental diets were supplemented with rapeseed oil, pork lard or palm oil at 60 g/kg. In the control diet, wheat starch was substituted for the fat source. The highest ileal digestibility of the fat and all FAs (except stearic acid) was observed in chickens fed lard. The content of fat in the breast meat of chickens was not significantly influenced by the fat supplements. The FA profiles of breast meat and abdominal fat reflected the FA composition of the diet. In the meat of chickens fed rapeseed oil, oleic acid was the predominant FA. Palmitic acid was the most abundant FA in the meat of chickens fed lard or palm oil. Oleic acid was the most abundant FA in the abdominal fat of all chickens. The highest mRNA expression of desaturases (Δ5-, Δ6- and Δ9-) was observed in chickens fed palm oil. The mRNA expression of hepatic FA synthase was higher in chickens fed palm oil or lard than in chickens fed rapeseed oil. The expression of HMG-CoA reductase was higher in chickens fed palm oil than in those fed rapeseed oil or lard. It can be concluded that rapeseed oil and lard are better sources of lipids than palm oil. These former two sources contain more digestible fatty acids and provide a lower concentration of SFAs in the meat and fat of chickens.

## Introduction

Supplemental fats are often used to increase dietary energy density to meet the requirements of fast-growing broiler chickens. Different fats and oils are usable in poultry nutrition: tallow, lard, palm fats and oils, vegetable oils, by-products of oil refining, and recovered frying oils [1]. Fats and oils contain fat-soluble vitamins, supply essential fatty acids, improve the palatability of feed, and reduce friction in feed mills. The major component of fats and oils is fatty acids (FAs). Fats and oils vary in their FA composition. The metabolism of fats depends primarily on the fatty acid (FA) profile. Tancharoenrat et al. (2014) [2] showed that unsaturated FAs were well digested in the lower ileum irrespective of the source of fat. The digestibility of saturated FAs (palmitic and stearic) was higher in a diet containing soybean oil (50 g/kg) than in a diet containing tallow (50 g/kg). A significant proportion of synthesized and absorbed saturated FAs (SFAs) are desaturated in tissues by desaturases to monounsaturated FAs (MUFAs). Fatty acid synthase produces saturated fatty acids (palmitate, stearate). Fatty acids are elongated and desaturated by enzymes in the endoplasmic reticulum membrane. For example, the conversion of stearoyl-CoA to oleyl-CoA is catalysed by a complex of three membrane-bound proteins:

$$Stearoyl‑CoA+NADH+H^{+}+O_{2}\to oleyl‑CoA+NAD^{+}+2H_{2}O$$

A variety of unsaturated fatty acids can be formed from oleate by a combination of elongation and desaturation reactions [3]. Kouba and Mourot (1998) [4] showed that polyunsaturated FAs (PUFAs) in the diet inhibited stearoyl-Δ9-desaturase in the liver, resulting in reduced conversion of SFAs to MUFAs. Increasing the content of linoleic acid (C 18:2) in the diet and in the hepatic tissue of broiler chickens decreased the desaturation of SFAs to MUFAs [5]. The FA profile of different tissues reflects the dietary FA profile [6]. Moreover, the partitioning of absorbed FAs is different in MUFAs and PUFAs. In chickens fed diets supplemented with four types of fat, MUFAs were higher in abdominal fat, whereas PUFAs were higher in muscle lipids [7]. Crespo and Esteve-Garcia (2002) [8] reported that broilers fed diets enriched with PUFAs deposited less abdominal and body fat than those fed SFAs or MUFAs, mainly due to the higher oxidation rate of PUFAs. The metabolism of FAs in birds and mammals is different. In mammals, most *de novo* synthesis of FAs occurs in adipose tissue, whereas in birds, most of the fat that is deposited in adipose tissue originates from lipid synthesis in the liver [9].

Rapeseed oil, animal fat, and palm fat are commonly added to feed mixtures for poultry. The public consumer is increasingly protesting against palm fat addition. The reasons are both ecological, in relation to the decline of forests, and nutritional, due to the high content of saturated fatty acids in palm fat. Pork lard also has a higher content of saturated fatty acids such as palm fat. Therefore, the objectives of the study were to compare the effect of different sources of fat on 1) the apparent ileal digestibility of fat and FAs, 2) the FA profiles of lipids in breast muscle and abdominal fat, 3) the mRNA expression of several lipid-related hepatic genes in broiler chickens.

## Materials and methods

### Birds and diets

The experiment was conducted on 240-day-old Ross 308 cockerels. The chickens were randomly allocated to four groups of 60 birds each and housed in pens (2 x 1.1 m) on wood shavings, with 20 chickens per pen. The experimental room was provided with gas heating and ventilation with a temperature-controlled fan. Each pen was equipped with 7 nipple drinkers, 3 pan feeders and a feed hopper. The temperature in the room at arrival was 32°C and gradually decreased to 20°C as the birds reached 28 days of age. The light cycle consisted of 16 h of light and 8 h of darkness. In the 1^st^ group (control), the fat supplement was substituted with wheat starch. The following fat sources were used: rapeseed oil (2^nd^ group), lard (3^rd^ group), and palm oil (4^th^ group). Rapeseed oil and refined palm oil were supplied by Fabio Produkt, Ltd. (Jičín, Czech Republic), and lard was purchased from a local shop. All supplements were added at 60 g/kg diet. Chromic oxide was mixed with the diets at 3.0 g/kg and used to measure the ileal digestibility of fatty acids. The chickens were fed one type of diet from the start to the end of the experiment (days 1–28 of age); the diets differed in the source of fat. Table 1 presents the composition and nutrient levels of the diets fed to the broiler chickens. The study protocol was approved by the Ethical Committee of the Institute of Animal Science. The experiment lasted from the 1^st^ to 28^th^ day of chicken life.

**Table 1:** Ingredients and analysed compositions of the diets without fat supplementation or supplemented with rapeseed oil, lard, or palm oil (g/kg).

Item	Control diet	Rapeseed oil	Pork lard	Palm oil
Wheat	529.5	529.5	529.5	529.5
Soybean meal	364.0	364.0	364.0	364.0
Calcium dihydrogen phosphate	14.6	14.6	14.6	14.6
Sodium chloride	3.0	3.0	3.0	3.0
Limestone (1–2 mm)	17.6	17.6	17.6	17.6
L-Lysine hydrochloride	1.3	1.3	1.3	1.3
DL-Methionine	0.5	0.5	0.5	0.5
L-Threonine	0.4	0.4	0.4	0.4
Vitamin-mineral premix^a^	5.0	5.0	5.0	5.0
Chromium oxide	3.0	3.0	3.0	3.0
Sodium carbonate	1.1	1.1	1.1	1.1
Rapeseed oil 00	0.0	60.0	0.0	0.0
Palm oil	0.0	0.0	0.0	60.0
Pork lard	0.0	0.0	60.0	0.0
Wheat starch	60.0	0.0	0.0	0.0
Dry matter	876.8	887.2	881.6	884.8
Calculated ME_N_ (MJ/kg)	12.19	13.23	13.17	13.19
Crude protein	228.1	228.8	222.1	231.3
Crude fat	18.5	70.7	70.8	70.9
Crude fibre	31.6	37.4	38.5	38.4
Ash	66.1	65.1	66.4	65.4
Calcium	10.25	10.19	10.20	10.22
Available phosphorus	4.54	4.55	4.52	4.48

^a^Vitamin-mineral premix provided per kg of diet: retinyl acetate, 3.6 mg; cholecalciferol, 13 μg; niacin, 40 mg; α-tocopheryl acetate, 50 mg; menadione, 3 mg; thiamine, 3 mg; riboflavin, 5 mg; pyridoxine, 4 mg; cyanocobalamin, 40 μg; calcium pantothenate, 12 mg; biotin, 0.15 mg; folic acid, 1.5 mg; choline chloride, 250 mg; ethoxyquin, 100 mg; iron, 50 mg; copper, 12 mg; iodine, 1 mg; manganese, 80 mg; zinc, 60 mg; and selenium, 0.2 mg.

### Sampling

At 28 days of age, all chickens were weighed. Five cockerels with an average live weight within the group were selected from each replication (15 birds/group) and slaughtered by decapitation. These chickens were eviscerated, and their livers were removed and stored at -70°C until further analysis. The left breast muscle (*M*. *pectoralis major*) was excised and stored at -18°C for later analysis. Abdominal fat pads were collected. The contents of the ileum between the Meckel's diverticulum and the ileo-caecal-colonic junction were collected and pooled for 3 birds to yield 5 samples per treatment. Samples were freeze-dried and used for the determination of the FA, fat, and Cr_2_O_3_ concentrations. The livers were excised, and approximately 0.2 g tissue samples were immediately submerged in RNAlater stabilization reagent (Qiagen, Germantown, MD, USA), followed by overnight storage at 4°C. The remaining liver tissue was kept at -20°C until analysis.

### Analyses

The feed was analysed as described previously [10]. Feed dry matter was determined by oven drying at 105°C, ash by ashing at 550°C [11], and fat by extraction with petroleum ether in a Soxtec 1045 apparatus (Tecator, Höganäs, Sweden). The crude fibre content was analysed according to AOAC official methods [12]. The crude protein content of the feed was assayed using a Kjeltec Auto 1030 (Tecator, Höganäs, Sweden). Feed calcium (Ca) and phosphorus (P) were determined after ashing of the samples. The total P in the diets was analysed using a vanadate-molybdate reagent [13]. The calcium content was determined by atomic absorption spectrometry using a Solar M6 instrument (TJA Solutions, Cambridge, UK). The phytate P contents of the diets were determined using a capillary isotachophoretic method [14]. Total lipids were extracted from the diets, hepatic tissue and breast muscle with 2:1 chloroform-methanol according to Folch et al. (1957) [15]. Alkaline trans-methylation of FAs was carried out according to ISO 5509. An HP 6890N gas chromatograph equipped with a programmed DB-23 capillary column (Agilent Technologies, Santa Clara, USA) was employed to determine the FA profile. The Cr content in ileal samples was determined by atomic absorption spectrometry using a Solaar M6 instrument (TJA Solutions, Cambridge, UK).

### RNA isolation and real-time PCR assay

Stabilized liver tissue samples (20 mg) were extracted for total cellular RNA using an RNeasy Mini Kit (Qiagen, Germantown, MD, USA) following the manufacturer’s instructions. Each sample was examined spectrophotometrically using a Nanodrop 1000 (Thermo Fisher Scientific, Waltham, USA) to estimate the RNA concentration and purity. Total RNA was used as a template for one-step PCR using a QuantiTect SYBR Green RT-PCR Kit (Qiagen, Germantown, MD, USA). The RT-PCR mixtures contained 5 μL of 2x QuantiTect SYBR Green RT-PCR Mastermix, 0.4 μL of QuantiTect RT Mix (Qiagen, Germantown, MD, USA), 0.3 μL of each forward and reverse primer (0.3 μM), and 1 μL of RNA template. The total RNA was diluted one hundred-fold to reduce possible inhibitory effects, and each reaction contained approximately 10–20 ng of RNA. The total volume was brought to 10 μL per reaction with an appropriate amount of RNase-free distilled water. The QuantiTect SYBR Green RT-PCR Kit contained ROX as a passive reference dye. Real-time PCR and data analysis were performed on 384-well plates using a 7900HT thermocycler (Applied Biosystems, Foster City, USA). The thermal cycling conditions were as follows: 50°C for 30 min for reverse transcription, followed by 95°C for 15 min and 40 PCR cycles with denaturing at 94°C for 15 seconds, annealing at 60°C for 30 seconds and elongation at 72°C for 15 seconds. Each sample was measured in triplicate. After PCR amplification, a melting curve analysis was performed. The following oligonucleotide primer pairs were used: beta actin (BACT, used as an internal control in all reactions, 5’-GAGGCAGCTGTGGCCATCT, 3’-AAATTGTGCGTGCATCAAGGA, accession number AY_045724), 3-hydroxy-3-methyl-glutaryl-coenzyme A reductase (HMG-CoA reductase, 5’-ATGATTTCAAGTTGTCGCACTCC, 3’-TAGGTCCTACATTTACCCTGGATG, accession number NM_204485), carnitine palmitoyltransferase 1 (5’-TGACGTCGATTTCTGCTGCT, 3’-GCAGCGCGATCTGAATGAAG) [16], fatty acid synthase (5’-CAATGGACTTCATGCCTCGGT, 3’-GCTGGGTACTGGAAGACAAACA) [17], Δ5-fatty acid desaturase (5’-GAGCCATCGGTGAGGGTTTC, 3’-CTCCAGTCCTTTCCTTGCGT) [16], Δ-9-fatty acid desaturase (5’-GGAGCCCTAGGAGAAGGTTTC, 3’-AAATTGAAGCGCCAGCCAAA) [16], Δ6-fatty acid desaturase (QuantiTect Primer Assay, QT01510824). All primers were synthesised by Generi Biotech Ltd. (Hradec Králové, Czech Republic), and the QuantiTect Primer Assays were purchased from Dynex Ltd. (Buštěhrad, Czech Republic). The 2^-ΔΔCT^ method was used for the quantification of all gene expression relative to internal control levels.

### Calculations

The pooled standard error of the mean (SEM) was calculated using the GLM procedure in the Statistical Analysis System (SAS 2001). Treatment effects were evaluated using one-way analysis of variance. All differences were considered non-significant at P > 0.05.

## Results

The source of fat in the diet did not influence the performance characteristics of the chickens. The average weight of the chickens in each group at the end of the experiment ranged from 1859 to 1895 g, and the average feed intake per chicken per day ranged from 88.5 to 91.7 g. The FA profiles of rapeseed oil, lard and palm oil are shown in Table 2. Table 3 presents the FA concentrations in the chicken diets. The highest concentrations of unsaturated FAs (both MUFAs and PUFAs) were present in the diet supplemented with rapeseed oil. In contrast, the highest concentration of SFAs was present in the diet supplemented with palm oil.

**Table 2:** Fatty acid composition (g/100 g FAs determined) of the rapeseed oil, lard and palm oil used as supplements in chicken diets.

Fatty acid		Rapeseed oil	Lard	Palm oil
Lauric	12 : 0	0.02	0.09	0.21
Myristic	14 : 0	0.33	1.35	0.99
Palmitic	16 : 0	6.65	21.60	37.94
Palmitoleic	16 : 1	0.46	2.18	0.60
Stearic	18 : 0	3.33	11.78	5.18
Oleic	18 : 1 c9	51.04	34.70	39.45
cis-Vaccenic	18 : 1 c7	2.56	2.20	-
α-Linoleic	18 : 2 n6	15.45	10.40	8.80
Linolenic	18 : 3 n3	7.05	0.73	0.28
Arachidic	20 : 0	0.45	0.21	0.34
Eicosenoic	20 : 1 n9	1.03	0.65	-
Eicosadienic	20 : 2 n6	0.06	0.45	-
Arachidonic	20 : 4 n6	-	0.84	-
Other FAs		11.32	12.50	6.07
Total SFAs		10.98	35.54	44.86
Total MUFAs		55.15	39.82	40.08
Total PUFAs		22.56	12.02	9.10

**Table 3:** Fatty acid concentrations (g/100 g) in chicken diets.

Fatty acids		Control diet	Rapeseed oil	Lard	Palm oil
Lauric	12 : 0	0.00	0.00	0.01	0.01
Myristic	14 : 0	0.03	0.00	0.08	0.06
Palmitic	16 : 0	0.51	0.53	1.17	1.79
Palmitoleic	16 : 1	0.02	0.02	0.10	0.02
Stearic	18 : 0	0.23	0.16	0.60	0.37
Oleic	18 : 1 c9	0.49	3.02	1.65	1.84
cis-Vaccenic	18 : 1 c7	0.03	0.17	0.11	0.05
Linoleic	18 : 2 n6	1.01	1.78	1.03	1.05
Linolenic	18 : 3 n3	0.11	0.52	0.12	0.10
Arachidic	20 : 0	0.01	0.01	0.01	0.02
Eicosenoic	20 : 1 n9	0.01	0.02	0.03	0.01
Eicosadienic	20 : 2 n6	0.00	0.00	0.02	0.02
Arachidonic	20 : 4 n6	0.00	0.00	0.01	0.00
Other FAs		0.03	0.05	0.07	0.01
Total SFAs		0.80	0.73	1.91	2.27
Total MUFAs		0.55	3.23	1.90	1.93
Total PUFAs		1.13	2.32	1.20	1.17

The ileal digestibility of fat in chickens fed a diet supplemented with wheat starch was significantly lower than that in chickens fed diets supplemented with rapeseed oil, lard and palm oil (Table 4). The highest ileal digestibility of all FAs (except stearic acid), SFAs, MUFAs and PUFAs was observed in chickens fed lard. Among the individual FAs, very high ileal digestibilities (>90%) were found for linoleic, α-linolenic and eicosadienic acid in chickens fed lard. The ileal digestibilities of lauric, palmitic, arachidic and arachidonic acid were not significantly different.

**Table 4:** Ileal digestibility of fat and FAs in chickens fed diets without fat supplementation or supplemented with rapeseed oil, lard, or palm oil (%).

Item		Control diet	Rapeseed oil	Lard	Palm oil	SEM
Fat		35.1^b^	68.7^a^	78.1^a^	57.8^a^	4.47
Fatty acids						
Lauric	12 : 0	80.8	83.8	89.0	78.1	1.52
Myristic	14 : 0	46.8^c^	63.7^bc^	86.2^a^	74.9^ab^	3.83
Palmitic	16 : 0	65.2	65.1	81.5	72.0	2.54
Palmitoleic	16 : 1	53.4^b^	60.8 ^b^	85.2^a^	57.1^b^	3.25
Stearic	18 : 0	50.2^b^	73.2^a^	71.8^a^	58.5^b^	2.66
Oleic	18 : 1 c9	72.3^b^	81.9^ab^	88.4^a^	74.6^b^	2.05
Linoleic	18 : 2 n6	86.9^ab^	89.2^ab^	93.2^a^	83.0^b^	1.25
α-Linolenic	18 : 3 n3	86.6^ab^	92.0^a^	93.3a	79.3^b^	1.51
Arachidic	20 : 0	59.5	69.6	72.3	61.1	2.81
Eicosenoic	20 : 1 n9	62.3^b^	77.2^a^	88.6^a^	57.7^b^	3.16
Eicosadienic	20 : 2 n6	69.4^b^	73.2^b^	91.1^a^	52.7^c^	3.17
Arachidonic	20 : 4 n6	43.7	56.6	46.8	47.2	1.82
SFAs		64.8^b^	78.1^a^	82.7^a^	62.1^b^	2.02
MUFAs		68.0^b^	78.6^ab^	84.2^a^	69.2^b^	1.96
PUFAs		65.7^b^	68.7^b^	80.8^a^	60.0^c^	1.66

^abc^ Values in the same row with different superscripts differ significantly at P < 0.05

The breast meat of chickens fed a diet supplemented with starch contained significantly less dry matter and crude protein than the meat of broilers fed diets supplemented with lard and palm oil (Table 5). The concentration of fat in the breast meat of chickens was not significantly influenced by the composition of the diet. The profiles of FAs in the breast meat were different in all treatment groups of chickens. The highest content of unsaturated FAs was present in the meat of chickens fed rapeseed oil, whereas the highest content of SFAs was in the meat of chickens fed a diet supplemented with palm oil. Palmitic acid was the most abundant FA in the meat of chickens fed diets supplemented with lard and palm oil. In the breast meat of chickens fed rapeseed oil, oleic acid was the predominant FA. Rapeseed oil significantly increased the content of FAs containing 20 carbon atoms in the chain, i.e., arachidic, eicosenoic, eicosadienic and arachidonic acids. Oleic acid was the most abundant FA in the abdominal fat of all chickens (Table 6). The abdominal fat of chickens fed rapeseed oil contained more unsaturated FAs than the abdominal fat of the other chickens.

**Table 5:** Chemical composition and fatty acid concentrations in breast meat in broiler chickens fed diets without fat supplementation or supplemented with rapeseed oil, lard, or palm oil.

		Control diet	Rapeseed oil	Lard	Palm oil	SEM
Dry matter (%)		22.4^b^	23.3^ab^	23.7^a^	23.9^a^	0.21
Crude protein (%)		20.1^b^	20.9^ab^	21.5^a^	21.5^a^	0.21
Fat (%)		3.45	3.56	3.45	3.81	0.17
Fatty acids (mg/100 g)							
Lauric	12 : 0	1.0^b^	1.9^a^	0.9^b^	2.0^a^	0.12
Myristic	14 : 0	14^b^	21^c^	14^b^	48^a^	2.7
Palmitic	16 : 0	341^c^	387^c^	441^b^	605^a^	25.7
Palmitoleic	16 : 1	71^a^	61^a^	34^c^	48^b^	3.2
Stearic	18 : 0	97^c^	137^b^	183^a^	143^b^	6.5
Oleic	18 : 1 c9	542^b^	853^a^	362^c^	522^b^	35.0
Linoleic	18 : 2 n6	213^b^	456^a^	143^c^	178^bc^	23.1
α-Linolenic	18 : 3 n3	21^b^	91^a^	11^c^	17^bc^	5.9
Arachidic	20 : 0	1.5^c^	3.7^a^	2.6^b^	2.5^b^	0.18
Eicosenoic	20 : 1 n9	9^b^	17^a^	5^c^	7^bc^	0.93
Eicosadienic	20 : 2 n6	12^a^	13^a^	7^b^	8^b^	0.61
Arachidonic	20 : 4 n6	37^b^	66^a^	28^c^	37^b^	2.9
Other FAs		87^b^	113^a^	68^c^	72^c^	6.5
Total SFAs		462^c^	558^c^	649^b^	810^a^	34.9
Total MUFAs		677^b^	988^a^	442^c^	614^b^	39.9
Total PUFAs		308^b^	675^a^	210^c^	266^bc^	34.1

^abc^ Values in the same row with different superscripts differ significantly at P < 0.05; NS—Not significant.

**Table 6:** Fatty acid concentrations (mg/100 g) in abdominal fat.

Fatty acids		Control diet	Rapeseed oil	Lard	Palm oil	SEM
Lauric	12 : 0	35^c^	45b^c^	63^b^	93^a^	5
Myristic	14 : 0	600^b^	545^b^	868^a^	851^a^	31
Palmitic	16 : 0	19222^b^	14234^d^	17661^c^	21644^a^	543
Palmitoleic	16 : 1	6565^a^	2751^c^	3589^b^	3724^b^	268
Stearic	18 : 0	4158^b^	4222^b^	5598^a^	4627^b^	144
Oleic	18 : 1 c9	33493^b^	40884^a^	30069^c^	35269^b^	836
cis-Vaccenic	18 : 1 c7	2311^a^	2203^a^	1914^b^	1644^c^	59
Linoleic	18 : 2 n6	9744^bc^	14379^a^	9356^c^	10405^b^	390
Linolenic	18 : 3 n3	1159^b^	4017^a^	853^c^	1130^b^	234
Arachidic	20 : 0	85^c^	126^a^	95b^c^	101^b^	4
Eicosenoic	20 : 1 n9	337^b^	590^a^	352^b^	317^b^	22
Eicosadienic	20 : 2 n6	116b^c^	128^b^	169^a^	97c	6
Arachidonic	20 : 4 n6	128^c^	210^a^	150^b^	164^b^	10
Other FAs		734a^b^	816^a^	689^b^	686^b^	45
Total SFAs		24239^b^	19362^c^	24518^b^	27514^a^	625
Total MUFAs		42920^b^	46541^a^	36064^c^	41097^b^	868
Total PUFAs		11528^bc^	19247^a^	10844^c^	12141^b^	634

^abcd^Values in the same row with different superscripts differ significantly at P < 0.05.

**Fig 1:**
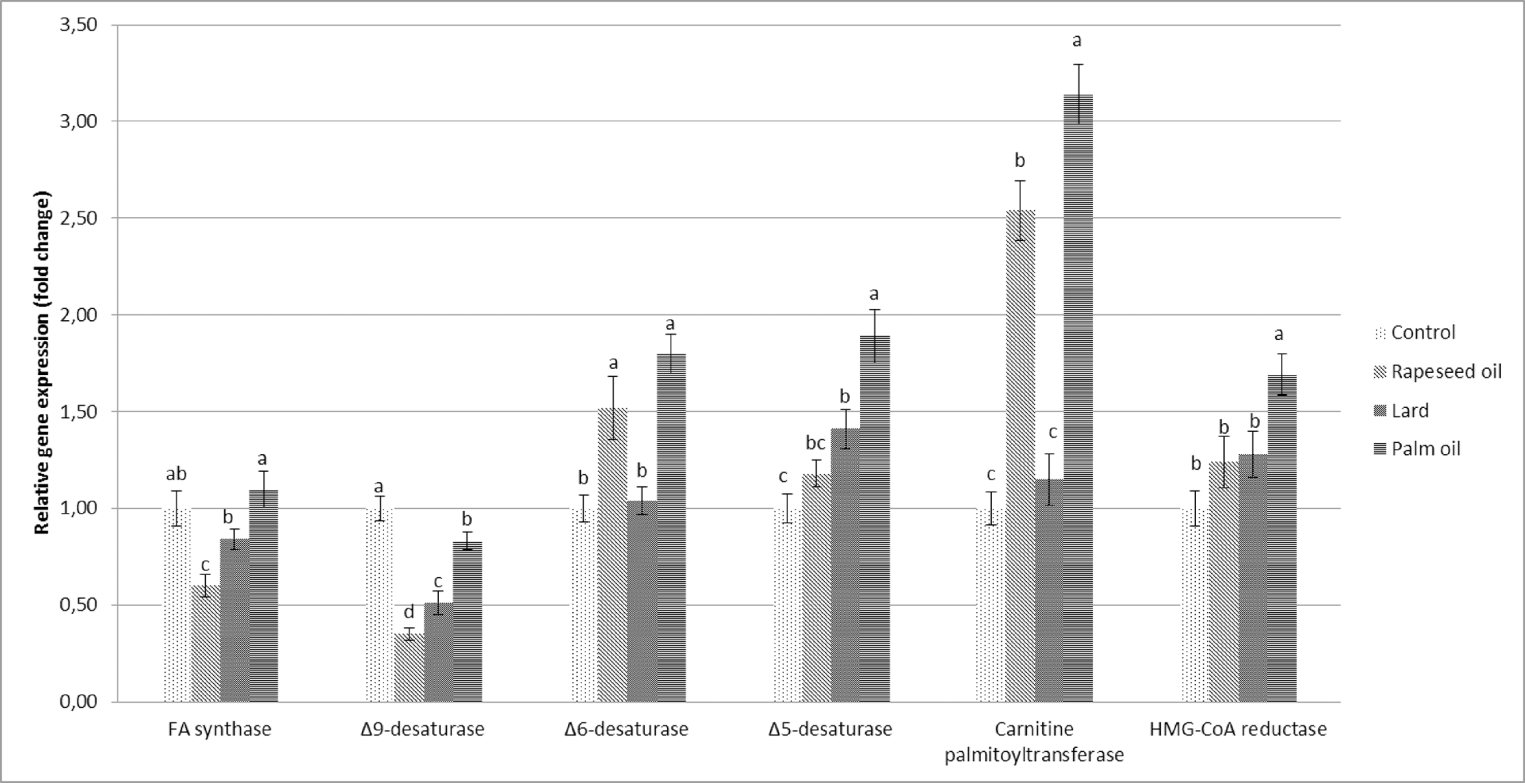
Relative mRNA expression of lipid-related hepatic genes in broiler chickens fed diets without fat supplementation or supplemented with rapeseed oil, lard, or palm oil. Fold change in gene expression normalized to an endogenous reference gene (β-actin) relative to the untreated control. Data are the mean ± SEM.

Fig 1 presents data on the relative mRNA expression of six hepatic genes related to lipid metabolism measured as RQ (2^-ΔΔCT^). The relative expression of carnitine-palmitoyltransferase was low in chickens fed diets supplemented with lard and high in chickens fed rapeseed or palm oil. The highest relative expression of HMG-CoA reductase was observed in chickens fed palm oil, while this expression was similar in chickens fed rapeseed oil or lard. Rapeseed oil reduced the expression of FA synthase. The three fat sources differed in their effects on the relative expression of desaturase genes. The highest expression of all desaturases was observed in chickens fed palm oil. All fat sources reduced the expression of Δ9-desaturase in comparison with that in the control.

## Discussion

The ileal digestibility of fat was low in chickens fed a diet without fat supplements. The reason for this low digestibility may be the low fat content in this diet. Because, the level of fat in the diet could be in proportion to the secretion of bile, which is involved in fat digestion. Tancharoenrat et al. (2014) [2] reported that unsaturated FAs were well digested irrespective of the source of fat. These authors found that the ileal digestibility of palmitic and stearic acids was higher in a diet containing soybean oil than in a diet containing tallow. In the present experiment, the ileal digestibility of all FAs (except stearic acid) was higher in chickens fed a diet enriched with lard than in chickens fed rapeseed or palm oil. The lowest ileal digestibilities of SFAs, MUFAs, PUFAs, and ten out of twelve FAs were found in chickens fed a diet supplemented with palm oil, which is consistent with the results of Tancharoenrat et al. (2014) [2].

There was no direct relationship between the FA digestibility and FA profile in tissues. The FA profile in breast meat was primarily affected by the FA profile of the feed. In abdominal fat, MUFAs dominated in all groups of chickens. This dominance may be related to the difference between structural and depot lipids [18].

Both breast meat and abdominal fat are edible parts of the chicken body. Information on the effects of FAs on human health is extensive [19, 20, 21]. Numerous studies have shown that dietary FAs strongly influence the tissue FA composition [6,18]. As expected, the FA profiles in the breast meat and abdominal fat reflected the FA composition of the fat supplement. The effect of rapeseed oil, which is rich in oleic and linoleic acids, on the content of unsaturated FAs in the breast meat was more pronounced than that of lard and palm oil. The concentrations of linoleic and arachidonic acids were highly correlated (*r* = 0.908; P < 0.001), suggesting a significant conversion of linoleic acid by Δ6-desaturase. This result also suggests a significant activity of FA elongase because the addition of two carbons was also necessary.

In chickens fed lard or palm oil, the relative proportions of PUFAs were 15–16% of the total FAs, which was less than that in the respective diets (24 and 22%). Oleic acid dominated the lipids deposited in the bodies of all chickens. This dominance may be related to the fact that the oleic acid content in tissue lipids depends not only on the oleic acid intake but also on *de novo* synthesis.

Evaluating the relationship between biochemical parameters and mRNA levels is one way to evaluate the relevance of gene expression data. Musa et al. (2007) [22] noticed that mRNA levels do not always correlate with the amount of functional protein produced. In our study, the high content of SFAs in palm oil was consistent with the high expression of mRNA from all hepatic desaturases. Delta-9 desaturase converts stearic acid to oleic acid, which is required for the synthesis of cell membranes [3]. Chickens desaturate SFAs to prevent a decrease in the unsaturated FA/SFA ratio below a certain level [23]. Delta-5 and Δ-6 desaturases are the rate-limiting enzymes and are crucial in PUFA-related pathways [24].

Sanz et al. (2000) [25] reported that the activity of FA synthase in the liver of chickens fed a sunflower oil-enriched diet was lower than the same activity in chickens fed a tallow-enriched diet. Dietary PUFAs apparently inhibit *de novo* FA synthesis. This is consistent with the results obtained in our experiment. The expression of FA synthase mRNA was higher in chickens fed palm oil or lard than in those fed rapeseed oil. HMG-CoA reductase is the rate-limiting enzyme of cholesterol synthesis. Its expression was higher in chickens fed a diet supplemented with palm oil or lard. This observation is consistent with the finding that the substitution of rapeseed oil for lard significantly decreased the cholesterol concentration in chicken breast muscle [10].

Carnitine palmitoyltransferase is a mitochondrial enzyme responsible for the transfer of activated FAs inside the mitochondria for β-oxidation. Literature data regarding the dietary effects on carnitine palmitoyltransferase activity are ambiguous. Smink et al. (2010) [5] reported that the hepatic activities of carnitine palmitoyltransferase were similar in chickens fed diets supplemented with sunflower oil or palm oil. Sanz et al. (2000) [25], however, found that the activity of carnitine palmitoyltransferase was higher in heart extracts of chickens fed a diet enriched with sunflower oil than in chickens fed a diet containing tallow. Preferential oxidation of PUFAs over SFAs in chickens was reported by Newman et al. (2002) [26]. In our study, the mRNA expression of this enzyme was low in the livers of chickens fed a diet supplemented with lard and high in the livers of chickens fed diets supplemented with rapeseed oil or palm oil.

## Conclusion

In this study, three fat supplements with different fatty acid profiles were compared in the diets of broiler chickens. The digestibilities of the fatty acids in rapeseed oil and lard were higher than that in palm oil. The use of rapeseed oil in the diets of broiler chickens provided the optimum composition of fatty acids in the breast meat and fat. The mRNA expression of genes associated with lipid metabolism is consistent with this opinion.
